# Which characteristics of written feedback are perceived as stimulating students’ reflective competence: an exploratory study

**DOI:** 10.1186/1472-6920-13-94

**Published:** 2013-07-08

**Authors:** Hanke Dekker, Johanna Schönrock-Adema, Jos W Snoek, Thys van der Molen, Janke Cohen-Schotanus

**Affiliations:** 1 Institute for Medical Education, University of Groningen and University Medical Center Groningen, A. Deusinglaan 1, FC40, 9713 AV, Groningen, the Netherlands; 2 Center for Research and Innovation of Medical Education, University of Groningen and the University Medical Center Groningen, Groningen, the Netherlands; 3 Department of Primary Care, University of Groningen and University Medical Center Groningen, Groningen, the Netherlands

**Keywords:** Undergraduate medical education, Written feedback, Reflective writing, Professional development

## Abstract

**Background:**

Teacher feedback on student reflective writing is recommended to improve learners’ reflective competence. To be able to improve teacher feedback on reflective writing, it is essential to gain insight into which characteristics of written feedback stimulate students’ reflection processes. Therefore, we investigated (1) which characteristics can be distinguished in written feedback comments on reflective writing and (2) which of these characteristics are perceived to stimulate students’ reflection processes.

**Methods:**

We investigated written feedback comments from forty-three teachers on their students’ reflective essays. In Study 1, twenty-three medical educators grouped the comments into distinct categories. We used Multiple Correspondence Analysis to determine dimensions in the set of comments. In Study 2, another group of twenty-one medical educators individually judged whether the comments stimulated reflection by rating them on a five-point scale. We used *t*-tests to investigate whether comments classified as stimulating and not stimulating reflection differed in their scores on the dimensions.

**Results:**

Our results showed that characteristics of written feedback comments can be described in three dimensions: *format of the feedback* (phrased as statement versus question), *focus of the feedback* (related to the levels of students’ reflections) and *tone of the feedback* (positive versus negative). Furthermore, comments phrased as a question and in a positive tone were judged as stimulating reflection more than comments at the opposite side of those dimensions (*t* = (14.5) = 6.48; *p* = < .001 and *t* = (15) = −1.80; *p* < .10 respectively). The effect sizes were large for *format of the feedback comment* (*r* = .86) and medium for *tone of the feedback comment* (*r* = .42).

**Conclusions:**

This study suggests that written feedback comments on students’ reflective essays should be formulated as a question, positive in tone and tailored to the individual student’s reflective level in order to stimulate students to reflect on a slightly higher level. Further research is needed to examine whether incorporating these characteristics into teacher training helps to improve the quality of written feedback comments on reflective writing.

## Background

Current views on learning and societal developments have led to a shift from knowledge-based to competency-based medical curricula [1-5]. The main focus of these curricula is on the development of competencies – demonstrable abilities encompassing knowledge, skills and professional behaviour. An underlying assumption is that a clear set of competencies can help students to self-direct their own learning. In other words, students can actively plan, monitor and evaluate their learning processes to enhance their professional development. For the development of these self-directed learning skills, reflection – a metacognitive process that creates greater understanding of self and situations to inform future action – is widely acknowledged as a crucial attribute [6-8].

Since reflection does not come naturally to most students [6,9], Aronson (2011) has suggested that formal education is required to enhance students’ reflective competence [10]. In medical education, various methods are in use to facilitate reflection: reflective storytelling and writing, critical incident analysis, writing personal development plans and portfolios [11-14]. Although these methods help most students to make sense of their experiences, the potential of reflection may not be fully realized without personal teacher/supervisor support [8,10]. A supportive mentor who provides feedback on students’ reflective assignments seems to be a prerequisite for enhancing students’ reflective competence [15]. We consider in particular written feedback valuable, since this kind of feedback is captured on paper and can be reread by students at a later time [16].

Teachers often perceive providing written feedback on reflective writing as a difficult task and some have expressed a need for training [17]. In order to fulfill their needs and to develop adequate teacher training courses on providing feedback on reflective writing, it is essential to gain insight into which characteristics of written feedback will help teachers to stimulate students’ reflection processes. Therefore, the aims of this study were to determine the characteristics of written feedback comments on students’ reflective writing assignments (Study 1) and to examine which of these characteristics are perceived as conducive to students’ reflection processes (Study 2).

## Methods

### Context and materials

Both studies were performed at the medical school of the University of Groningen, the Netherlands. The pre-clinical Bachelor’s program of this medical school lasts three years. Each study year consists of four 10-week Problem Based Learning modules including tutorial groups and a Professional Development module which spans the academic year. This Professional Development module is aimed at encouraging students to reflect on their professional behaviour and their first practical experiences. For their reflection on professional behaviour, students make use of assessment forms gathered at the end of each 10-week module. Each student is evaluated on his professional behaviour by their tutor on the hand of an assessment form that focuses on 3 dimensions: Task Performance, Aspects of Communication, and Personal Performance [18]. Tutors rate each student per dimension on a scale ranging from 1 (poor) to 10 (excellent). This quantitative mark needs to be accompanied by a qualitative narrative. Each student is also assessed at the end of every module by two peers who use similar assessment forms. Furthermore, the students meet – once per five weeks under supervision of a teacher – in small groups (10 students per group) as part of the Professional Development module. During these sessions, students do not only learn to reflect on their assessments of professional behaviour in the tutorial groups, but also on different experiences gained during short internships at a general practitioners office or during an outpatient clinic, for instance their first patient-related encounters. Halfway through the academic year, after collecting the various assessment forms, each student writes a reflective essay in which he or she summarizes the judgements obtained, reflects on differences between these judgements, determines major improvement points and describes an action plan to improve his or her future behaviour. The student puts the reflective essay together with the assessment forms and other completed assignments in a portfolio and hands it in to the teacher. To ensure that the feedback was given timely – which is essential to effective delivery of feedback [19] – the teachers provide written feedback on the various assignments in the students’ portfolios within two weeks after receiving the portfolio. In order to help students to enhance their professional development, the written feedback comments that the teachers provide should stimulate reflection. For our study, we made use of teachers’ written feedback comments (n = 43) on students’ reflective essays.

### Study 1 - Determining characteristics of written feedback comments

#### Participants

Twenty three medical educators (teachers and educational scientists) were asked to participate in this study, which was aimed at determining which characteristics can be distinguished in written feedback comments. They were conscientiously selected on the basis of their knowledge and skills. They were all involved in the Professional Development course in the bachelor phase of the undergraduate medical programme as developer and/or supervisor and therefore they formed an important stakeholder group. All participants were instructed about the procedure of writing reflective essays and trained in general didactics on providing feedback. Furthermore, they all have been active, as participant or as trainer, in workshops on how to stimulate reflection on experience for instance by applying Korthagen’s ALACT (Action, Looking back, Awareness, Creating alternatives and Trial) –model [20]. Participants were informed about the purpose of the study and participation was voluntary. The data were processed confidentially.

#### Analysis and procedure

To determine the characteristics of the written feedback comments, we used a nonlinear variant of Principal Components Analysis, called Multiple Correspondence Analysis (MCA). MCA is an analysis method which yields outcomes based on the frequency with which concepts or variables are associated with each other. MCA has been widely used in, for instance, marketing research [21,22] and is suitable for addressing our research question. Compared to, for instance, the Delphi method or the Q-method, the advantage of MCA is that each participant contributes equally to the end result and that there is little risk of drop-out as the participants need to make an effort only once. MCA summarizes the most apparent relationships between nominal variables. The nominal variables in this study were the 43 written feedback comments. MCA can be used to identify the structure in a data set, i.e. detect underlying dimensions in our written feedback comments. The accompanying procedure involves having the comments sorted into categories by individual raters. Therefore, we gave each participant all 43 comments – each printed on a different paper card – and asked them to sort the comments (individually) into distinct categories based on similarities that they observed between the comments. We informed them that there were no right or wrong solutions and that they could make as many categories as they felt necessary. The only requirement was that a category had to contain at least two cards.

Two important aspects for determining which number of dimensions provides the best fit are 1) the inertia and 2) the interpretability [23]. The inertia refers to the amount of variance explained. Per dimension, inertia can range from 0.0 to 1.0. All dimensions of a MCA solution should be interpretable, as a solution that is not interpretable and theoretically logical is of little value [24-26]. Usually, up to three dimensions are retained [23]. Since statistical experts suggest investigating the interpretability of several solutions to ensure selection of the solution that makes the most sense and displays the most scientific sensibility [27,28], we decided not to restrict ourselves to a maximum of three dimensions, but investigate the interpretability of up to four dimensions. To optimize the interpretation process, investigator triangulation was applied. The first author and two co-authors independently interpreted the dimensions of each solution and subsequently discussed their interpretations to reach consensus on the interpretation of the dimensions and the best solution. MCA was performed with SPSS (version 18.0.3).

### Study 2 - Comments that stimulate reflection

#### Participants and procedure

We asked 21 experts, Dutch or Belgian medical educators, at an invitational conference on reflection to participate in this second study. They were all engaged in professional development programmes in their own institutes in the Netherlands or Belgium and were interested in further education concerning how to optimize students’ reflection skills. We asked this ‘convenience’ sample to rate the extent to which each feedback comment *stimulates reflection* or *not* on a five-point Likert-type scale, ranging from *not at all* (− −) to *very well* (++). Participants were informed about the purpose of the study and participation was voluntary and anonymous.

#### Analysis

After a research team discussion consensus was reached to use 75% as a cut-off percentage. This percentage is also a generally accepted ‘rule of thumb’ within our country. If 75% or more of the participants of the expert panel assessed a particular comment as stimulating reflection (+ and ++) it is considered as stimulating reflection. A comment was considered as not stimulating reflection if 75% or more of the participants assessed a comment as not stimulating reflection (− and --). The comments that did not satisfy either of these conditions were labelled neutral. We performed independent *t*-tests to determine whether comments classified as stimulating reflection differed from those classified as not stimulating reflection with respect to their scores on the dimensions found in study 1. We calculated the effect size (*r*) to find out whether differences were relevant, with the thresholds for small, medium and large effects being *r* = .10, *r* = .30 and *r* = .50, respectively [29].

#### Ethical statement

National practice in the Netherlands, where this study was carried out, does not require ethical approval for educational studies and surveys. However, in this study we adhered to the following ethical principles. The researchers had no hierarchical relationship with the participants. Participation was voluntary and data were processed either anonymously (study 2) or at least confidentially (study 1). Furthermore, in accordance with the university privacy policy, all materials derived from the portfolios were anonymized. This means that none of 1) the students from whose portfolios the feedback comments were derived, 2) the teachers who provided the feedback comments or 3) the participants in our studies are identifiable from the data, with the result that no possible harm can arise from publication.

## Results

### Characteristics of written feedback comments

The outcomes of both the MCA and the interpretation process indicated that the three-dimensional solution was the best solution for describing the characteristics of written feedback comments on students’ reflective writing. The inertia of the first two dimensions were good (.728 and .560 respectively) and the inertia of the third dimension satisfactory (.377). All three dimensions were clearly interpretable, with the dimensions being interpreted as *format of the feedback comment*, *focus of the feedback comment* and *tone of the feedback comment* (Table 1). At one end of the dimension *format of the feedback comment*, the items were formulated as questions, for example ‘Elaborate: what about your role in the group? Why do you want it to change? Is the poor preparation of your fellow students caused by language problems?’ (comment 43), while items at the opposite pole were more formulated as a statement. An example of such a statement is ‘An adequate essay’ (comment 41). Items on the dimension *focus of the feedback comment* represented comments aimed at completing the descriptive aspect of the reflective essay versus comments that go more deeply into the content of the reflection, thus touching on higher levels of reflection. The former comments relate to the layout of the essay, missing information or unsatisfactory elaboration of the learning points, for example ‘Self-assessment is lacking; how are you going to work out your learning goals?’ (comment 16). Feedback comments that concerned the content of the essay often contained a suggestion to improve future professional behaviour, for example ‘You’re a hard worker, but in the group you could push yourself more to the fore, show that you have an opinion’ (comment 30). Items on one pole of the dimension *tone of the feedback comment* reflected a positive environment in which the feedback was given, for example ‘You are a good group member, stimulating too. But in your reflective essay you mention a lack of interest. What makes you think that?’ (comment 25). Comments on the opposite pole represented remarks on shortcomings, for example ‘It’s a pity that your content is shallow, it is the bare minimum, something already commented on by your coach as a point for improvement’ (comment 29). The internal consistencies of these three dimensions were high (α = .98, .96, and .93, respectively).

**Table 1:** **Object scores of a three**-**dimensional solution and inertia**

**Written feedback comment**		**Format**	**Focus**	**Tone**
1.	How do you plan to deal with dominant people?	1,806	-,665	,265
2.	You have six years of medical school to go and I have a lot of confidence in how you will develop. But beware; a strong point can become a pitfall. For example, wanting to do everything perfectly can lead to a burnout.	,359	1,022	1,027
3.	How are you going to work out your learning goals?	2,303	−1,655	-,078
4.	Great essay, clear learning goals.	−1,222	−1,227	,779
5.	I find your reflective essay recognizable.	−1,009	-,978	,420
6.	You’re self-critical and you pick up on the remarks of others very well.	-,642	-,259	,906
7.	Okay.	−1,169	−1,160	,254
8.	Almost right, but how are going to realize your learning goals?	2,032	−1,349	,365
9.	The most important point in your reflective essay is missing, the self-reflection.	-,356	,146	−1,510
10.	Clear and precise.	−1,221	−1,231	,743
11.	I feel you have diagnosed your strengths and weaknesses clearly. Particularly the fact that you noticed that you hold back in discussions and thus relinquish the opportunity to lead the discussion in another direction.	-,113	,946	1,082
12.	Your accent is music to my ears.	-,465	,208	-,845
13.	Adequate essay with clear learning goals.	−1,223	−1,234	,678
14.	You picked up on your own learning goals well, you’re smart enough but some more self-discipline would be desirable.	,098	1,371	,899
15.	How are you going to work on your negative points?	2,303	−1,655	-,078
16.	Self-assessment is lacking, how are you going to work out your learning goals?	1,968	−1,345	-,367
17.	I could not find your self-evaluation form; your self-reflection essay is too brief.	-,483	-,024	−1,349
18.	Good that you share your assessors’ points of criticism but I don’t see this properly reflected in the points for improvement. They are there if I read between the lines, but you should try to be more specific.	,484	,658	,369
19.	Good essay with adequate content, structure and use of language. The self-assessment and the reflective essay are also good.	-,985	-,654	,562
20.	An adult response to criticism.	-,891	-,636	,831
21.	You seem well able to imagine how others value you; your learning goal is interesting. A hint: consciously experiment with a pitfall. If you change your role in the group, the role of others in the group changes also.	,428	1,493	1,469
22.	The evaluation was well reflected upon and formulated into clear learning goals.	-,895	-,920	,738
23.	Assessment forms are missing, the assignments are neatly produced; you are active in the group and a stimulating person.	-,328	,753	,142
24.	It’s striking that your essay is the longest I received. You are a feisty lady who has a tendency to cut corners a bit too often. Spend some more time on reflection and your assignments and you’ll be fine.	,270	1,407	,958
25.	You are a good group member, stimulating too. But in your reflective essay you mention a lack of interest. What makes you think that?	1,108	,705	1,358
26.	Try to formulate more concisely - although your language skills are good, your texts are too long.	-,021	,950	−1,584
27.	The self-assessment form is missing, but you worked it out OK in the reflective essay.	-,605	,088	-,503
28.	A more extensive reflection than others.	-,815	-,588	-,259
29.	It’s a pity that your content is shallow, it is the bare minimum, something already commented on by your coach as a point for improvement.	,131	,534	−1,669
30.	You’re a hard worker, but in the group you could push yourself more to the fore to show that you have an opinion.	,193	1,523	,284
31.	An assessment form is missing, you have a pleasant manner with the patients and they like you.	-,300	,810	-,107
32.	A nice summary to learn from.	−1,010	-,939	,258
33.	Please pay more attention to the following: careful language use, professional language use and discipline. The reflective essay is unprofessional.	,240	,598	−1,661
34.	Be on time!	-,156	,194	−2,047
35.	Pay a bit more attention to the layout.	-,303	,202	−2,641
36.	Reveal more of yourself in your essay.	,302	,282	−1,431
37.	Just like everyone else, this is mostly a summary of other people’s feedback.	-,610	,043	−1,396
38.	You dare to be critical and you support this reasonably (which is good). But being self-critical is also important. Sometimes you seem very pleased with yourself, and if you get feedback you often point the finger at others. At other times you seem perfectly well prepared to notice the same about yourself.	,167	1,473	,667
39.	Some of the assessment forms are missing! You come across as very convinced of yourself, which is all very well and good but you should also show flexibility and display a genuine interest in others, which you do to some extent but not completely. Therefore, listen harder; you already know what you’re going to say yourself. P.S. good time management and punctuality.	,260	1,645	,660
40.	Written clearly, in keeping with my earlier remarks, but with evident progress made.	-,688	-,136	,291
41.	An adequate essay.	−1,221	−1,237	,642
42.	Good essay, it shows that you have thought it over. Putting less into discussions is not the same as being more moderate. Stick to your guns but learn to control your timing.	,348	1,556	,579
43.	Elaborate: what about your role in the group? Why do you want it to change? Is the poor preparation of your fellow students caused by language problems?	1,929	-,717	,298
Inertia		.728	.560	.377

### Comments stimulating reflection

Of the 43 feedback comments, eleven were classified as stimulating reflection (comments 3, 8, 15, 16, 18, 21, 25, 38, 39, 42, 43) and 6 as not stimulating reflection (comments 5, 7, 12, 35, 40, 41). Comments that were rated as stimulating reflection differed significantly from those rated as not stimulating reflection on the dimension *format of the feedback comment*, (*t*(14.5) = 6.48; *p* < .001) and marginally on *tone of the feedback comment* (*t*(15) = −1.80; *p* < .10). The effect sizes were large for *format of the feedback comment* (*r* = .86) and medium for *tone of the feedback comment* (*r* = .42). Closer inspection revealed that comments that were rated as stimulating reflection were predominantly phrased as questions and were phrased in a more positive tone. No differences were found regarding the dimension *focus of the feedback comment*.

## Discussion

The main goal of written feedback on students’ reflective writing is to stimulate and improve students’ reflection skills in order to enhance their professional development.

Our study revealed three dimensions characterizing written feedback comments on students’ reflective essays: *format of the feedback comment* (questions versus statements), *focus of the feedback comment* (related to the levels of students’ reflections) and *tone of the feedback comment* (positive versus negative). Besides, we found that comments perceived as stimulating reflection were predominantly formulated as questions and tended to be phrased in a positive tone.

The results of our study are partly in line with the more general feedback literature. This literature indicates that, in general, feedback has two main functions: to inform students about a certain performance and/or to actively stimulate them to improve their performance [30]. The *format of the feedback* dimension relates to these functions: written feedback comments formulated as statements correspond with the informing function and comments formulated as questions relate to the improvement function of feedback. If students are supposed to improve their reflection skills, written feedback comments on their reflective writing should preferably be formulated as a question. Furthermore, it is known from literature that a positive affective climate is crucial to the learning process and helps enhancing the impact of feedback [31,32]. This corresponds with our finding that comments considered as stimulating reflection were mainly phrased in a positive tone. A possible explanation may be that feedback on a negative tone can raise resistance within students [33,34].

We did not find differences between comments perceived as stimulating and as not stimulating reflection on the dimension *focus of the feedback comment*. Unlike the former two dimensions, this dimension seems to be more specific to reflective writing rather than related to the general feedback literature. In the literature on reflection, several levels of reflection are described, evolving from descriptive writing to critical reflection, where students explore and critique assumptions and also show emotional insight [7,35,36]. We noticed that the quality of the reflective essays of our students differed, with some students only describing experiences and others really attempting to reflect on their experiences. It appears that feedback comments on all levels of reflection can stimulate reflection. One could surmise that students whose reflective writing is still at the lowest level of descriptive writing can benefit from feedback on their description, while others who really critically analyse the remarks about their professional behaviour, benefit more from feedback on their reflection. According to this line of reasoning, feedback comments can stimulate refection all along the dimension *focus of feedback*.

The main goal of providing written feedback comments was to enhance students’ reflection on their professional development. Based on our participants’ perceptions, we presume that this goal may be achieved by formulating feedback comments on students’ reflective writing as a question and in a positive tone. Considering that educational literature indicates that challenging students to perform on higher levels may help to increase their skills [31,37], this goal may even better be served if the comments focus on a reflection level that is slightly above the level on which the student performs. In the literature on reflection, different levels of reflective writing are described, ranging from descriptive writing to critical reflection [36]. Future research might investigate which kinds of questions can be asked to challenge students towards reflection levels slightly above the level on which they perform and examine the effectiveness of challenging students towards higher reflection levels.

A limitation of our study is that we did not include students in our study to find out which types of comments stimulate reflection. We intentionally chose to start this area of research with experienced medical educators. To move the field forward and examine the hypotheses generated through our qualitative work that feedback on reflective writing should be formulated a) in a question, b) positive in tone and c) on a reflection level slightly above that of the student, future research might try to investigate effectiveness of feedback differing in these characteristics. For instance, an experiment might be designed, in which students are given different types of feedback and instructed to revise their reflection after feedback. The outcomes might shed more light on the effectiveness of type of feedback in terms of improvement in reflective narratives. In this way, the field may get beyond qualitative and opinion data.

A second limitation is that only about 25% of the comments were regarded as feedback that stimulates reflection. However, despite the low numbers in our analyses, we did find significant differences between comments considered as stimulating and not stimulating reflection and these differences seemed relevant considering the effect sizes that we found.

Our 3 dimensions *format*, *focus* and *tone* of feedback may provide useful starting points for teacher training. Early experiences in teacher training sessions focusing on these characteristics are positive. The three dimensions seem to provide our teachers with a feasible framework for providing written feedback on students’ reflective writing. Future research should focus on the effect of this training on the quality of feedback comments. Does the quality of feedback comments of teachers who are trained with this conceptual framework improve? And, linked to that, do students who receive feedback comments, (1) tailored to their reflection levels, (2) formulated as questions to lift them to slightly higher reflection levels, and (3) formulated in a positive tone, improve their reflective writing?

## Conclusions

This study showed that written feedback comments on students’ reflective essays can be characterized in terms of *format*, *focus* and *tone* of feedback. In addition, our study indicates that written feedback comments should be formulated as a question, positive in tone and tailored to the individual student’s reflective level in order to stimulate students to reflect on a slightly higher level. Further research is needed to investigate the effectiveness of incorporating these three dimensions into teacher training to improve the quality of written feedback comments on reflective writing.

## Competing interest

The authors declared that they have no competing interest.

## Authors’ contribution

All authors contributed to the conception and design of the study. HD was responsible for the acquisition and interpretation of the data, and for writing and revising the manuscript. JSA, JS, TvdM and JCS contributed to the critical revision of the manuscript. All authors approved the final version of the manuscript.

## Authors’ information

Hanke Dekker, Msc is an Educationalist at the Institute for Medical Education at the University of Groningen and University Medical Center Groningen, The Netherlands. She is involved in the design and organization of the Professional Development programme.

Johanna Schönrock-Adema, PhD, is a Researcher at the Center for Research and Innovation of Medical Education at University of Groningen and the University Medical Center Groningen, The Netherlands.

Jos W. Snoek, MD, PhD, Neurologist, is a Professor in Clinical Education and Director of the Master program of Medical Education Institute, University of Groningen and University Medical Center Groningen, the Netherlands.

Thys van der Molen, MD, PhD, is a Professor of Primary Care Medicine and Coordinator of the Professional Development programme at the University of Groningen and University Medical Center Groningen, The Netherlands.

Janke Cohen-Schotanus, PhD, is a Professor in Medical Education and Head of the Center for Research and Innovation of Medical Education, University of Groningen and University Medical Center Groningen, The Netherlands.

## Pre-publication history

The pre-publication history for this paper can be accessed here:

http://www.biomedcentral.com/1472-6920/13/94/prepub
